# Prevalence of ocular trauma in 6–12-year-old children living in Shahroud, Iran

**DOI:** 10.1186/s12886-022-02541-5

**Published:** 2022-07-26

**Authors:** Hassan Hashemi, Reza Pakzad, Mehdi Khabazkhoob, Abbasali Yekta, Mohammad Hassan Emamian, Akbar Fotouhi

**Affiliations:** 1 Noor Research Center for Ophthalmic Epidemiology, Noor Eye Hospital, Tehran, Iran; 2 Department of Epidemiology, Faculty of Health, Ilam University of Medical Sciences, Ilam, Iran; 3 Department of Basic Sciences, School of Nursing and Midwifery, Shahid Beheshti University of Medical Sciences, Tehran, Iran; 4 Department of Optometry, Mashhad University of Medical Sciences, Mashhad, Iran; 5 Ophthalmic Epidemiology Research Center, Shahroud University of Medical Sciences, Shahroud, Iran; 6 Department of Epidemiology and Biostatistics, School of Public Health, Tehran University of Medical Sciences, Tehran, Iran

**Keywords:** Ocular trauma, Cross-sectional study, Prevalence, Children, Iran

## Abstract

**Aim:**

To determine the prevalence of ocular trauma in Iranian children aged 6–12 years.

**Methods:**

This population-based cross-sectional study, comprised the first phase of the Shahroud Schoolchildren Eye Cohort Study on primary school children using cluster sampling in urban areas and census in rural areas. The students underwent the measurement of uncorrected and corrected visual acuity as well as non-cycloplegic, cycloplegic, and subjective refraction. The history of trauma, hospitalization, and surgery due to trauma was collected from parents using a questionnaire.

**Results:**

The data of the trauma history was recorded for 5267 out of 5620 students. The mean age of the students was 9.7 ± 1.7 years (range: 6–12 years), 53.7% of them were boys, and 79.3% were from urban areas. A positive history of ocular trauma was found in 285 participants, and the lifetime prevalence of ocular injury (95% CI) was 5.2% (4.6–5.9). Blunt trauma was the most common ocular injury with a prevalence of 66.2%. There was a significant positive assocation between ocular trauma and living in rural areas (OR: 1.49, p: 0.012), older age (OR: 1.17, *p* < 0.001), and male sex (OR: 1.62, p: 0.002). Furthermore, 9.3% and 4.7% of the traumas required hospitalization and surgical intervention, respectively.

**Conclusion:**

This study found a marked prevalence of ocular trauma compared to previous studies. Male sex, older age, and living in rural areas were associated with ocular trauma, which could be due to differences in lifestyle preference, outdoor exposure, and dangerous situations. Educational programs and safety instructions should be encouraged.

## Introduction

Ocular injuries are a major public health problem such that it is one of the leading causes of monocular vision loss in the world [1] and visual morbidity in children [2]. Recent studies indicate that 24 million people suffer from eye injury in the US, of whom 1.5 million have visual impairment due to eye injury and 147,000 are total blind due to eye injury [3]. Ocular injury patients need to be hospitalized due to vision loss [4] such that the overall rate of hospitalization for individuals below 20 years is 8.9 in 100,000 in the US [5] and the incidence of eye injuries requiring hospitalization is 15.2 in 100,000 in Australia [6].

The magnitude of the effect of ocular trauma ranges from slight injuries to severe injuries and vision loss [7]. These injuries, in addition to the health burden, are associated with marked socioeconomic consequences for the patient, family, and society. Studies conducted in the United States showed that the annual direct treatment costs of ocular injuries were more than $8 million in 1989 [8], which increased to more than $88 million in 2006 [5]. This is while ocular injuries impose direct costs of more than $155 million on the health system annually in other countries including Australia [6], which will certainly increase considering indirect costs including lost productivity by young men and need for caring facilities[8].

Several studies have estimated the prevalence or incidence of ocular injuries [5–19]. The results of these studies showed a higher cumulative lifetime prevalence of ocular injuries in the elderly [12] and a higher incidence in adults and children [14]. However, most of these studies were conducted in the setting of hospitals and clinics [5–12] and few studies have been carried out in the general population [13–17]. Although the majority of ocular injuries occur in individuals below 17 years and the highest hospitalization rate is seen in children below 15 years [6], most of the studies have been conducted in older adults. On the other hand, the pattern of ocular injuries has shifted from the workplace to home [9], indicating that studies conducted in the settings of hospitals or workplaces, which are mostly done on older adults, may not reflect the true situation. Therefore, there is a marked gap in the epidemiology of ocular injuries in children using studies with a population-based design.

The epidemiology of ocular injuries in Iranian children is not clear yet. Researchers have carried out several hospital-based studies to investigate ocular injuries in Iran [10], but they may not reflect the true status of the society due to the setting in which they were conducted. Hashemi et al. [23] conducted a population-based study known as the Tehran Eye Study to estimate the prevalence of ocular injuries but it was performed in subjects above 40 years. Since no information is available on the prevalence of ocular injuries in Iranian children and the available studies in this regard were mostly conducted in certain populations rather than the general population, this study was performed to determine the prevalence of ocular trauma in children aged 6–12 years living in Shahroud.

## Methods

This study was conducted on the data of the phase one of Shahroud Schoolchildren Eye Cohort Study that was carried out in 2015 [24]. The target population of this study was the children living in Shahroud. Cluster (classrooms) sampling was done in urban areas while census sampling was applied in rural areas due to their small populations. In urban areas, 200 out of 473 classrooms were systematically selected as clusters. Further details regarding sampling and methodology are presented elsewhere [24].

First, non-cycloplegic auto refraction was done for all students using the Nidek ARK-510A auto refractokeratometer. Next, if a student wore spectacles, spectacle-corrected visual acuity was measured and lensometry was done. Then, all students underwent the measurement of uncorrected visual acuity using the Nidek CP-770 chart projector at three meters and the autorefraction data were refined using retinoscopy (Heine Beta retinoscope, HEINE Optotechnic, Hersching, Germany). For all students, the examinations were first done in the right eye and then in the left eye. Subjective refraction was done in students whose visual acuity was not 20/20. Finally, cycloplegic refraction was done using 1% cyclopentolate drops.

The parents completed a questionnaire including questions on the history of trauma, its type, and medical services required following ocular trauma. History of trauma was recorded as sharp, blunt, and chemical trauma. Moreover, trauma cases that resulted in hospitalization or surgery were also recorded. The patients who were not aware of the history of trauma were excluded from analysis. The data of 15 household assets was used to determine the economic status and an asset index was generated using principal component analysis and divided to low, middle, and high tertiles.

The prevalence of different types of ocular trauma in at least one eye as well as the prevalence of hospitalization due to trauma is reported as percentage and 95% confidence interval. If the prevalence was too low or too high, a binomial distribution was used to calculate the confidence interval. Simple and multiple logistic regression analysis were used to evaluate the assocation between ocular trauma and some risk factors (crude and adjusted). The effect of cluster sampling was considered for standard error calculation. Moreover, post stratification adjustment was done to Apply the sampling weights. P values less than 0.05 were considered significant.

### Ethical issue

This study was conducted according to the tenets of the Helsinki Declaration and its protocol was approved by the Ethics Committee of Shahroud University of Medical Sciences. Informed consent was obtained from the parents and oral consent was taken from the participants.

## Results

Of 6624 invited subjects, 5620 participated in the study (response rate = 84.8%, mean age = 9.7 ± 1.7, age range = 6–12 years). The data of trauma history was recorded for 5267 participants, which was used for analysis. Moreover, 2828 subjects (53.7%) were boys and 4178 (79.3%) lived in urban areas.

The total prevalence of ocular injuries was 5.2% (95% CI: 4.6–5.9). Among ocular injuries, blunt, sharp, and chemical trauma comprised 66.2%, 14.3%, 2.o%, of all injuries, respectively. The types of trauma were not report by 17.5% of participants who had history of trauma.

Table 1 presents the prevalence of ocular injury and its type in the study population and according to different variables. The prevalence of blunt, sharp, and chemical trauma was 3.4% (95% CI: 2.9—4.0), 0.7% (95% CI: 0.6—1.0), 0.1% (95% CI: 0.0—0.2), respectively.

**Table 1:** Prevalence of different types of ocular trauma by age, sex and living place, Shahroud, Iran, 2015

Variables		Prevalence (%) and 95% confidence intervals (in parentheses)			
		**Ocular Trauma**	**Blunt Trauma**	**Sharp Trauma**	**Chemical Trauma**
**Gender**	**Male**	6.2 (5.4 to 7.2)	3.9 (3.3 to 4.7)	1.1 (0.8 to 1.5)	0.1 (0.0 to 0.4)
	**Female**	3.9 (3.2 to 4.9)	2.9 (2.2 to 3.7)	0.3 (0.2 to 0.7)	0.1 (0.0 to 0.3)
**Residence place**	**Urban**	5.0 (4.3 to 5.7)	3.3 (2.8 to 3.9)	0.7 (0.5 to 1.1)	0.1 (0.0 to 0.2)
	**Rural**	7.1 (5.5 to 9.1)	4.6 (3.4 to 6.2)	0.7 (0.4 to 1.4)	0.2 (0.1 to 0.7)
**Age group (Year)**	**6**	2.9 (1.2 to 6.5)	1.7 (0.6 to 5.0)	0.3 (0.1 to 1.8)	––
	**7**	3.5 (2.3 to 5.2)	2.4 (1.5 to 3.7)	0.3 (0.1 to 1.1)	0.1 (0.0 to 0.4)
	**8**	4.2 (3.1 to 5.5)	2.4 (1.6 to 3.5)	0.8 (0.4 to 1.7)	0.1 (0.0 to 0.8)
	**9**	5.2 (3.8 to 7.0)	2.9 (2.1 to 4.0)	1.2 (0.6 to 2.1)	0.3 (0.1 to 1.0)
	**10**	5.4 (4.0 to 7.2)	3.9 (2.7 to 5.7)	0.5 (0.2 to 1.4)	–-
	**11**	6.3 (4.8 to 8.3)	4.5 (3.2 to 6.4)	0.9 (0.4 to 1.9)	0.1 (0.0 to 0.4)
	**12**	7.7 (5.7 to 10.3)	5.5 (3.7 to 8.0)	0.7 (0.3 to 1.8)	–-
**Total**		5.2 (4.6 to 5.9)	3.4 (2.9 to 4.0)	0.7 (0.6 to 1.0)	0.1 (0.0 to 0.2)

Table 2 presents eye care utilization following ocular trauma in children. Of 285 subjects with a positive history of ocular trauma, 104 (37.4%) were examined by an ophthalmologist and 26 (9.3%) were hospitalized. Only 12 subjects (4.7%) required surgical interventions following ocular trauma.

**Table 2:** Eye care utilization following ocular trauma in children, Shahroud, Iran, 2015

Events	Number ^*^	Percent (95% CI)
Ocular trauma in all children	285	5.2 (4.6 to 5.9)
Visited by physician for trauma	104	37.4 (31.6—43.6) ^#^
Admitted in hospital for trauma	26	9.3 (6.1—14.0) ^#^
Surgery for trauma	12	4.7 (2.6—8.5) ^#^

^*^ Because some category had missing; the total number is not same

^#^ percent calculated among children with history of ocular trauma; *CI* Confidence Intervals

Table 3 presents the results of simple and multiple logistic regression. According to the results of the multiple model, there was a significant positive assocation between ocular trauma and living in rural areas (OR: 1.49; 95% CI: 1.09 to 2.04), age (OR: 1.17; 95% CI: 1.08 to 1.23), and male sex (OR: 1.62; 95% CI: 1.23 to 2.12). No assocation was found between ocular trauma and economic status.

**Table 3:** Result of simple and multiple logistic regression for associations between of ocular trauma and their determinants

Independent variables		Simple logistic regression		Multiple logistic regression	
		**OR (95%CI)**	***p*-value**	**OR (95%CI)**	***p*-value**
Residence Place (Urban = 0)		1.45 (1.06 to 1.98)	0.019	1.49 (1.09 to 2.04)	0.012
Sex (Girl = 0)		1.62 (1.22 to 2.13)	0.001	1.62 (1.23 to 2.12)	0.002
Age (year)		1.17 (1.08 to 1.26)	< 0.001	1.17 (1.08 to 1.23)	< 0.001
Economic Status (Low = 0)	Middle	1.14 (0.84 to 1.55)	0.385	1.18 (0.87 to 1.61)	0.291
	high	1.11 (0.80 to 1.55)	0.524	1.10 (0.78 to 1.57)	0.605

*OR* Odds Ratio, *CI* Confidence Intervals

The mean ± SD of astigmatism was -0.39 ± 0.54 and -0.38 ± 0.51; in children with and without ocular trauma, respectively. Moreover, the mean ± SD of visual acuity (LogMar) was -0.06 ± 0.13 and -0.07 ± 0.09 in children with and without ocular trauma, respectively. In total 1.75% and 1.02% of children with and without ocular trauma had amblyopia, respectively. There were no statistically differences between two groups in terms of astigmatism (*p* = 0.741), visual acuity (*p* = 0.745), and amblyopia (*p* = 0.341).

## Discussion

Despite the importance of ocular trauma as a cause of blindness in children across the world [1, 2], its occurrence pattern is different in the world due to differences in environmental, cultural, and lifestyle [2]. Therefore, developed countries have made efforts to complete its epidemiological profile but there is little information from developing countries. Our extensive search only showed one population-based study about ocular trauma in Iran [23]. Therefore, the results of the present study can help to complete this information puzzle.

It should be noted that the majority of the previous studies on ocular trauma were hospital-based studies [5–12] and the estimated prevalence and incidence cannot be extrapolated to the general population due to methodological problems. There are a limited number of population-based studies in this regard, which are summarized in Table 4 [4–17].

**Table 4:** Prevalence of ocular trauma in population-based studies around the world

Author	Year	Study/Place	Age (Year)	Sample size	Prevalence, % (95% CI)	Common type
Keel et al. [15]	2017	Non-Indigenous Australians	50–98	3098	2.0 (1.6 – 2.6)	NR
		Indigenous Australians	40–92	1738	4.0 (3.2 – 5.1)	NR
Dandona et al. [13]	2000	Indian Urban Population	More than 15	2522	3.97 (2.52 – 5.42)	NR
Krishnaiah et al. [27]	2005	Indian Rural Population	More than 15	7771	7.5 (7.0 – 8.1)	NR
Hashemi et al. [23]	2011	Iranian Urban Population	1–96	4565	13.3 (12.0 – 14.5)	blunt
McCarty et al. [16]	1999	Australians Urban Population	More than 40	3260	19.4 (18.1 – 21.0)	Sharp Sharp
		Australians Rural Population	More than 40	1456	25.1 (22.2 – 27.9)	
Loon et al. [20]	2009	Singapore; Malay Eye Study	40–80	3264	5.1 (4.1 – 6.0)	Sharp
Jun et al. [28]	2015	Handan Eye Study/China	More than 30	5837	2.1 (1.8 – 2.5)	Sharp
Nirmalan et al. [17]	2004	Aravind Comprehensive Eye Survey/ India	More than 40	5150	4.45 (3.90 – 5.05)	Blunt
Wong et al. [29]	2000	Beaver Dam Eye Study	43–86	4909	19.80 (18.70 – 20.95)	Sharp
Vats et al. [30]	2008	Urban Population/ India	More than 16	6704	2.4 (2.0 – 2.7)	Blunt
Current study	2015	Shahroud / Iran	6 – 12	5267	5.2 (4.6 – 5.9)	Blunt

*CI* Confidence Intervals, *NR* Not reported

Keel et al. studied subjects above 40 years and estimated a prevalence of 2% in non-Indigenous and 4% in Indigenous Australians. [15] McCarty et al. conducted another study in individuals above 40 years in Australia and reported a prevalence of 21.1% for ocular trauma. [16] Studies conducted on subjects aged 15 and over in India showed a prevalence of 10.6% in rural regions [27] and 3.97% [13] and 2.4% [30] in urban areas. The prevalence of ocular trauma was 5% in the age group 40–80 years in Singapore Malay Eye Study [20] and 19.8% in the age group 43–86 years in the Beaver Dam Eye Study [29]. Hashemi et al. estimated a prevalence of 13.3% for ocular trauma in the age group 1–96 years in Tehran. [23] This is while the prevalence of ocular trauma was 5.2% in the present study. Caution should be exercised when comparing the results of the present study with previous studies due to the marked difference in the age range of the participants since all previous studies were conducted in adults and older age groups. The role of other factors such as cultural features and prevalence occupations should be considered too [13].

The results of the present study also showed that blunt trauma was the most common type of ocular trauma comprising 66% of the cases followed by sharp trauma (14.3%) and chemical trauma (2%). The majority of the previous hospital-based and population-based studies also found that blunt trauma was the most common type of ocular trauma which is due to more contact with objects like wood, stone, etc. However, some previous studies found chemical trauma [10–37] or sharp trauma [4] as the most common type. It seems that this difference is related to the study design or study population.

The present study found a significant positive assocation between age and the occurrence of ocular trauma, which is in line with previous studies [13]. However, it should be noted that since the lifetime prevalence of ocular trauma was estimated in all of the previous studies, it is expected to increase with age. Nonetheless, some studies reported a reduction in the lifetime prevalence of ocular trauma with age [20], which seems to be due to cohort effect and differences in the exposure of different generations to the risk factors of ocular trauma [16]. Moreover, a higher prevalence was expected in studies conducted in older age groups compared to the present study, while such studies [13] found a lower prevalence. Some studies attributed this finding to recall bias and under reporting in older subjects [30]. Nonetheless, attention should be paid to the role of other factors including differences in the age structure, prevalent occupations, and certain conditions of each society in terms of exposure to ocular trauma risk factors [32].

**Fig. 1:**
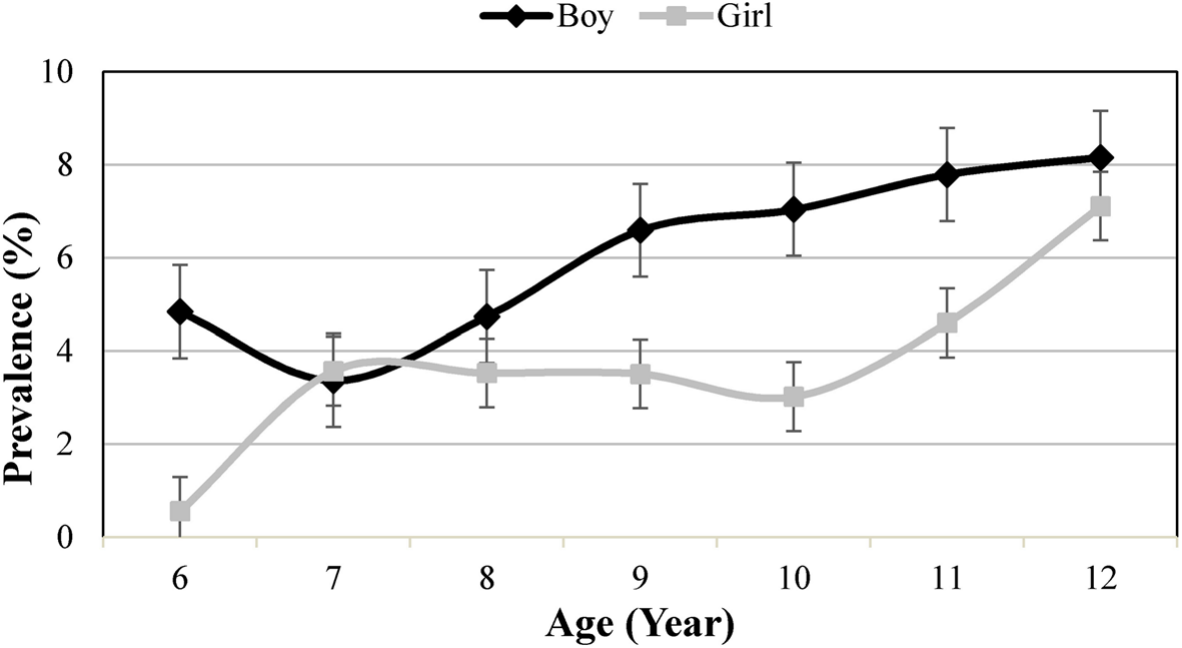
Prevalence of ocular trauma history in different age and sex group. Midpoint of each line shows the prevalence estimate and error bars indicate 95% confidence interval

In line with the results of other population-based studies [13], this study found a higher occurrence of ocular trauma in boys than in girls such that the odds of the occurrence of ocular trauma was 62% higher in boys. The higher prevalence of ocular trauma in boys was independent of age such that except for the age group 7 years, the prevalence was higher in all other age groups in boys (Fig. 1). It seems that the difference in the occurrence of ocular trauma is due to differences in lifestyle preferences and more outdoor exposure in boys [13]. Several hospital-based studies have reported a higher hospitalization rate in boys [5]. Keel et al. found more vision loss due to ocular trauma in boys versus girls, [15] indicating more severe ocular traumas in boys probably due to the higher frequency of more aggressive behavior in boys. Thylefors et al. conducted a review study and found that the inter-gender difference disappeared after the age of 70 years due to the similarity of lifestyle and occupational patterns. [32]

In line with studies conducted in Brazil [38], Australia [39], Turkey [40], and India [13], we expected a higher prevalence of ocular trauma in people with a worse economic state due to worse environmental conditions and lifestyle, but this association was not found in the present study or the Singapore Malay Eye Study [20]. The present study also found a higher prevalence of ocular trauma in rural areas than in urban areas, which is in line with previous studies [13]. It seems that differences in the lifestyle, economic factors, and awareness level on the one hand [36] and more exposure to dangerous occupations on the other hand^42^ are the reasons for this finding.

We also assessed the effect of ocular trauma on some visual parameters such as visual acuity, astigmatism, and amblyopia. Although we expected trauma to affect these parameters [15, 21, 41], we did not observe a significant difference in these outcomes between children with and without a history of ocular trauma. The difference between our results and other studies in this regard may be due to the design of current study (community-based), with most other studies (clinic or hospital-based), or the low prevalence of serious sharp and chemical traumas in the present study.

A large sample size, conducting examinations under strict supervision, and a high participation rate were strong points of this study. However, the observed assocation cannot be considered a causal assocation due to the presence of confounding factors, cohort effect, and cross-sectional design of the study. Moreover, the data used for this report, represents the population of Shahroud only (which is a relatively small city in Iran) and results may also be affected by Shahroud population lifestyle and environmental factors.

In conclusion, the prevalence of ocular trauma was 5.19% in the present study with blunt trauma being the most common type. The prevalence of ocular trauma was higher in boys than in girls and in rural regions compared to urban areas. The prevalence of ocular trauma also increased with age, which was due to cumulative calculation of the prevalence. The prevalence of ocular trauma was higher compared to similar study; therefore, it is important to include educational programs on safety instructions to decrease the risk of ocular injury, especially in school-age children.

## Data Availability

The data will be available in case of reasonable request by corresponding author. As described in the method section, this article is based on the data of the Shahroud Schoolchildren Eye Cohort study, which has thousands of variables. Considering that not all the outcomes of this study have been reported yet, currently, we do not have a policy of public access to the dataset of this study, but any researcher can receive the data related to the published articles by sending an email to corresponding author (emamian@shmu.ac.ir).

## Abbreviations

SSCECS: Shahroud Schoolchildren Eye Cohort Study
SD: Standard deviation
95% CI: 95% Confidence interval
